# Influences of Interface Properties on the Performance of Fiber-Reinforced Asphalt Binder

**DOI:** 10.3390/polym11030542

**Published:** 2019-03-22

**Authors:** Yinghao Miao, Ting Wang, Linbing Wang

**Affiliations:** 1 National Center for Materials Service Safety, University of Science and Technology Beijing, 30 Xueyuan Road, Haidian District, Beijing 100083, China; miaoyinghao@ustb.edu.cn (Y.M.); s20161201@xs.ustb.edu.cn (T.W.); 2 Department of Civil and Environmental Engineering, Virginia Tech, Blacksburg, VA 24061, USA

**Keywords:** fiber reinforced asphalt, interface property, surface energy, shear strength

## Abstract

This paper presents an experimental study about the influence of interfacial properties on the performance of fiber-reinforced asphalt. In this study, four types of fiber including one fiber-reinforced plastic (FRP), two lignin fibers, and one basalt fiber are used, and also four types of asphalt: Asphalt No. 90, asphalt No. 70, one styrene-butadiene-styrene (SBS) modified asphalt, and asphalt rubber are used. The surface energy parameters of various asphalts and fibers and the shear strength of various fiber-reinforced asphalts are measured. On the basis of these measurements, the influences of surface properties of asphalt and fiber on the performance of fiber-reinforced asphalt are analyzed. The results show that the shear strength of asphalt binder can be significantly increased by adding fibers, and the reinforcement effect is closely related to the types of asphalt and fiber. It was discovered, for the same asphalt, that the basalt fiber has the best reinforcement effect, followed by the two lignin fibers, and the FRP. For the same fiber, asphalt rubber was the most reinforced, followed by the SBS modified asphalt, asphalt No. 70 and asphalt No. 90. It was also discovered, for the same asphalt, the higher the surface energy of the fiber, the better the fiber reinforcement effect. The analysis indicates a good correlation between the work of adhesion between asphalt and fiber and the effect of fiber reinforcement. The results can be used as a basis for the selection of the proper fiber-asphalt combination to improve fiber reinforcement effects.

## 1. Introduction

Improving the performance of asphalt binder and asphalt mixture has always been an important research target in pavement engineering. By adding a certain amount of fiber into the asphalt mixture, the performance of the asphalt mixture can significantly improve and the service life of the asphalt pavement can be prolonged. Fiber-reinforced asphalt mixtures are widely used in different grades of roads because of their excellent performance and because of their reasonable cost [1,2,3,4,5]. With the development of technology, the types of fiber used to reinforce asphalt mixtures are increasing. Many fibers such as asbestos, lignin, polymer, glass, and basalt fibers are used for asphalt mixture to improve performance [6,7,8].

Many researchers have also investigated and evaluated the performance and the material design of fiber-reinforced asphalt mixture. Tapkin [3] studied the performance of polypropylene fiber-reinforced asphalt mixture by laboratory test. Chen et al. [7] used polyester, polyacrylonitrile, lignin, and asbestos fibers to compare the volume characteristics and mechanical properties of different fiber-reinforced asphalt mixtures. Xu et al. [9] evaluated the performance of polyester, polyacrylonitrile, lignin, and asbestos fiber-reinforced asphalt under the influence of temperature and moisture. Khattak et al. [4] studied the rheological properties of carbon nanofiber modification on asphalt. Their results showed that the addition of carbon nanofiber can improve the viscoelastic response of asphalt mixture and the rutting resistance. Yoo et al. [5] quantitatively analyzed the contribution of fibers to the tensile strength of fiber-reinforced asphalt using a direct tensile test and a bond strength estimation method. They found that the contribution of fibers to the tensile strength of fiber-reinforced asphalt was about 25.5%. Qian et al. [10] investigated the pullout strength of fibers using a multiple-fiber pullout test. Gibson et al. [11] analyzed the fatigue cracking resistance of fiber-reinforced asphalt mixture using an indoor test and a full scale accelerated test. Giustozzi et al. [12] studied the application of fiber reinforcement in a high reclaimed asphalt pavement (RAP) content warm asphalt mixture. Kamaruddin et al. [13] compared the water sensitivity of ordinary asphalt mixtures with fiber-reinforced asphalt mixtures by laboratory tests. Polypropylene and polyester fibers were selected in the tests. The results showed that the water sensitivity of the mixtures was reduced and the water damage resistance of the mixtures was increased by the addition of fiber. Klinsky et al. [14] studied the performance of polypropylene and aramid fiber-reinforced asphalt mixture against water damage, fatigue damage, and cracking by laboratory tests. The mechanical properties, such as dynamic modulus, were also analyzed. The results show that using these two fibers can enhance the performance of asphalt pavement comprehensively. Wang et al. [15] conducted research on the fatigue properties of polyacrylonitrile fiber-reinforced asphalt mixture by laboratory tests, and a fatigue model of fiber-reinforced asphalt was proposed.

The above research shows that fiber-reinforced asphalt has remarkable mechanical advantages in performance as compared with normal asphalt. However, most of the studies are focused on the macro-performance. Due to the complexity of the types of fiber used in different studies [16,17,18,19,20,21], it is difficult to find some generalized conclusions. At the same time, research on the mechanism of fiber-reinforced asphalt is relatively limited, and there are still many problems to be solved.

In the study presented in this paper, the performance of fiber-reinforced asphalt is analyzed from the perspective of asphalt-fiber interface. Four kinds of fiber and four kinds of asphalt are selected in the study, and the surface characteristics of various asphalts and fibers are measured, respectively. The fall cone test is used to evaluate the shear strength of different fiber-reinforced asphalts to characterize their performance. On this basis, the influences of surface properties of fiber and asphalt on the performance of reinforced asphalt are analyzed, which provides a reference for the material selections of fiber-reinforced asphalt.

## 2. Materials and Methods

### 2.1. Materials

**Figure 1:**
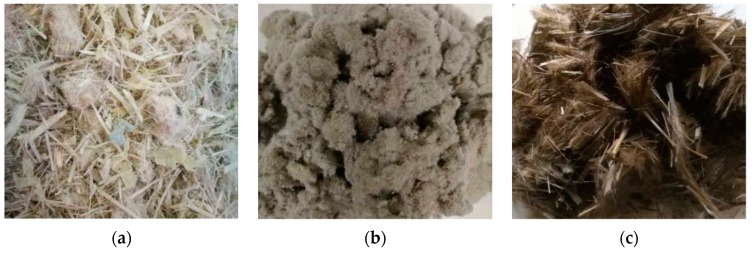
Selected fibers: (**a**) fiber-reinforced plastic (FRP); (**b**) lignin fiber; (**c**) basalt fiber.

In this study, four types of fiber and four types of asphalt were selected for experimental evaluations. The test fibers included one fiber-reinforced plastic (FRP), two lignin fibers, and one basalt fiber. Figure 1 shows the typical fibers for the test. The test asphalts included the asphalt No.90, the asphalt No.70, one styrene-butadiene-styrene (SBS) modified asphalt, and one asphalt rubber.

### 2.2. Test Methods of Interface Properties

The surface energy of asphalt was tested in solid state. Equation (1) shows the relationship between contact angle and surface energy [22]. If the contact angle of some reference liquids with known surface energy parameters on the asphalt surface is obtained by test, the surface energy of the asphalt can be calculated by a linear regression in accordance with Equation (1). In this study, the contact angle of asphalt was tested at 25 °C by the sessile drop method using a SL150 optical contact angle goniometer (USA KINO Industry CO. Ltd., Boston, MA, USA). The selected reference liquids were deionized water (laboratory preparation), ethylene glycol (Sinopharm Chemical Reagent Co., Ltd., Shanghai, China), glycerol (Sinopharm Chemical Reagent Co., Ltd., Shanghai, China), and formamide (Sinopharm Chemical Reagent Co., Ltd., Shanghai, China). Their surface energy parameters are listed in Table 1.

(1) $$\frac{\left(1+cos\theta \right)\gamma_{L}}{2\sqrt{\gamma_{L}^{d}}}=\sqrt{\gamma_{S}^{p}}\times \sqrt{\frac{\gamma_{L}^{p}}{\gamma_{L}^{d}}}+\sqrt{\gamma_{S}^{d}}$$

where θ is the contact angle of the liquid on the solid surface, $\gamma_{L}$ is the surface energy of the liquid, $\gamma_{L}^{d}$ is the dispersive component of surface energy of the liquid, $\gamma_{L}^{p}$ is the polar component of surface energy of the liquid, $\gamma_{S}^{d}$ is the dispersive component of surface energy of the solid, and $\gamma_{S}^{p}$ is the polar component of surface energy of the solid.

According to the Washburn equation (Equation (2)) and the van Oss–Chaudhury–Good Equation (Equation (3)), the surface energy of the fibers was measured by the capillary rise method (also called the Washburn method) by crushing the fibers into powder [23,24].

(2) $$\frac{L^{2}}{t}=\frac{\gamma_{L}Rcos\theta }{2\eta }$$

where L is the penetration distance, t is the penetrated time, $\gamma_{L}$ is the surface tension of the liquid, R is the effective pore radius, η is the dynamic viscosity of the liquid, and θ is contact angle. And

(3) $$\frac{\gamma \left(1+cos\theta \right)}{2}=\sqrt{\gamma_{S}^{LW}\gamma_{L}^{LW}}+\sqrt{\gamma_{S}^{+}\gamma_{L}^{-}}+\sqrt{\gamma_{S}^{-}\gamma_{L}^{+}}$$

where $\gamma_{S}^{LW}$ is the apolar or dispersive (Lifshitz–van der Waals) component of surface tension, $\gamma_{S}^{+}$ and $\gamma_{L}^{+}$ are Lewis acid components of surface tension of solid and liquid phases respectively, and $\gamma_{S}^{-}$ and $\gamma_{L}^{-}$ are Lewis base components of surface tension of solid and liquid phases respectively.

Four reference liquids with known surface energy parameters, i.e., positive-ethane (Sinopharm Chemical Reagent Co., Ltd., Shanghai, China), diiodomethane (Sinopharm Chemical Reagent Co., Ltd., Shanghai, China), toluene (Sinopharm Chemical Reagent Co., Ltd., Shanghai, China), and trichloromethane (Sinopharm Chemical Reagent Co., Ltd., Shanghai, China) were selected for the capillary rise test. The surface energy parameters of the reference liquids are listed in Table 2. On the basis of the test results as well as Equation (2) and Equation (3), the components of surface energy of solid powders, $\gamma_{S}^{LW}$, $\gamma_{S}^{+}$, and $\gamma_{L}^{+}$ were calculated. Then, the polar (Lewis acid-base) component ($\gamma_{S}^{AB}$) of surface energy of solid powders was calculated according to Equation (4), where the surface energy of solid $\gamma_{S}$ was obtained by Equation (5).

(4) $$\gamma_{S}^{AB}=\sqrt{\gamma_{S}^{+}\gamma_{S}^{-}}$$

(5) $$\gamma_{S}=\gamma_{S}^{LW}+\gamma_{S}^{AB}$$

On the basis of the test results of surface energy of asphalt and fiber, the work of adhesion of asphalt-fiber interface was calculated according to Equation (6) [25].

(6) $$W_{AF}^{D}=2\sqrt{\gamma_{A}^{d}\gamma_{F}^{d}}+2\sqrt{\gamma_{A}^{p}\gamma_{F}^{p}}$$

where $W_{AF}^{D}$ is the work of adhesion of asphalt-fiber interface without water, $\gamma_{A}^{d}$ and $\gamma_{A}^{p}$ are the dispersive and polar components of surface energy of the asphalt respectively, and $\gamma_{F}^{d}$ and $\gamma_{F}^{p}$ are the apolar or dispersive component ($\gamma_{F}^{LW}$) and polar components ($\gamma_{F}^{AB}$) of surface energy of the fiber respectively.

### 2.3. Test Method of Shear Strength of Asphalt Binder

**Figure 2:**
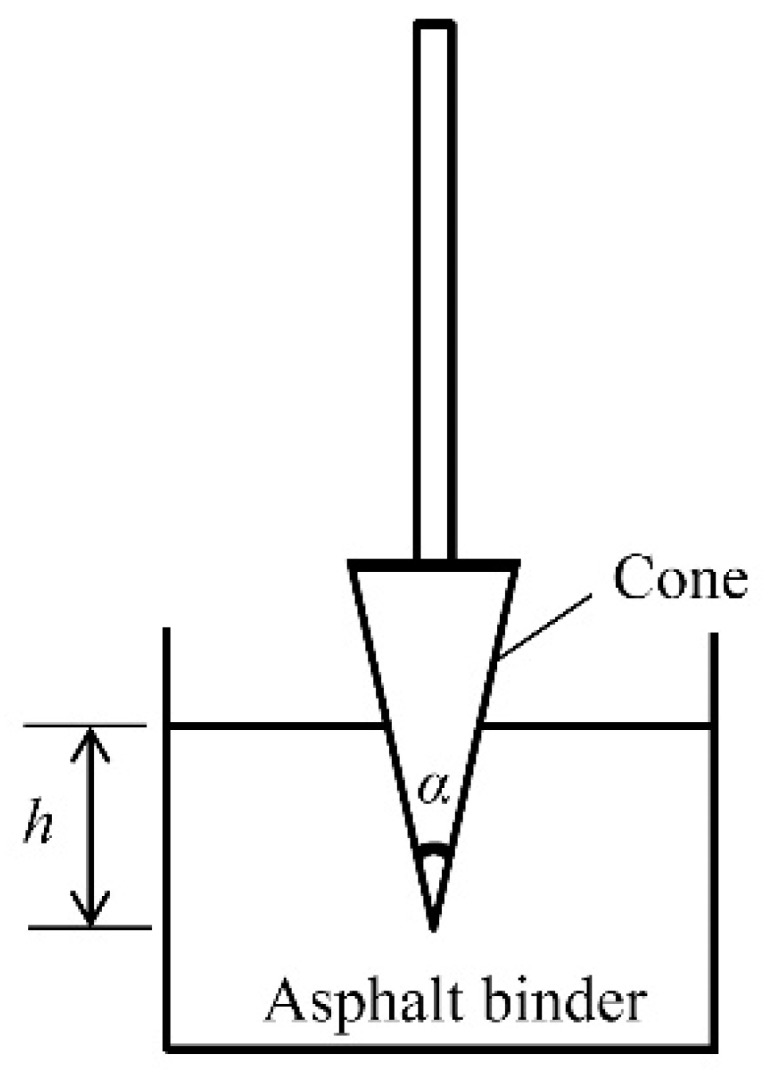
Sketch of the fall cone test.

The shear strength of asphalt binder was tested by the fall cone method using a SC-145 mortar consistency meter (Beijing Zhongke Lujian Testing Equipment Co., Ltd., Beijing, China). Figure 2 shows a schematic view for the test. The shear strength of asphalt binder was calculated by recording penetration depth for a certain period of time according to Equation (7) [26,27].

(7) $$\tau =\frac{Wcos^{2}\left(\frac{\alpha }{2}\right)}{\pi h^{2}\tan\left(\frac{\alpha }{2}\right)}$$

where W is the weight of the cone, h is the penetration depth, and α is the angle at the cone apex.

In this experiment, the cone with a mass of 0.5 kg and the cone apex angle of 30 degrees were used. The penetration depth corresponding to 10 s was used to calculate the shear strength of asphalt binder.

## 3. Test Results

### 3.1. Interface Properties

The contact angles of reference liquids on asphalt binder surface are measured by the sessile method at 25 °C. Each reference liquid is tested eight times at different positions on each asphalt surface. The average value is taken as the test result. The contact angles between four reference liquids and four asphalt binders are listed in Table 3. The surface energy and its components for various asphalts are calculated based on the contact angle test results, as listed in Table 4. Among the four asphalts, asphalt rubber has the greatest surface energy, followed by SBS modified asphalt, asphalt No.70, and asphalt No.90. The surface energy of the fibers is measured by the capillary rise method, and the results are listed in Table 5. The surface energy of basalt fiber is the largest among the four kinds of fiber, followed by lignin fiber A, lignin fiber B, and FRP. Based on the surface energy test results of asphalts and fibers, the work of adhesion of asphalt-fiber interface is calculated, where the results are listed in Table 6. Among them, the work of adhesion between asphalt rubber and basalt fiber is the largest, and the one between asphalt No.90 and FRP is the smallest.

### 3.2. Shear Strength of Asphalt Binder

**Figure 3:**
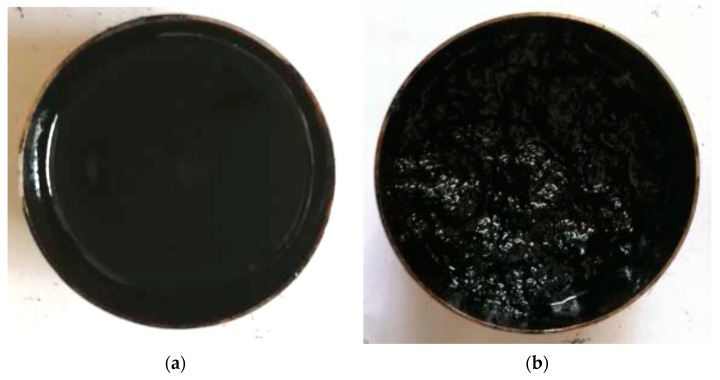
Typical surface of fiber-reinforced asphalt: (**a**) normal; (**b**) agglomeration.

**Figure 4:**
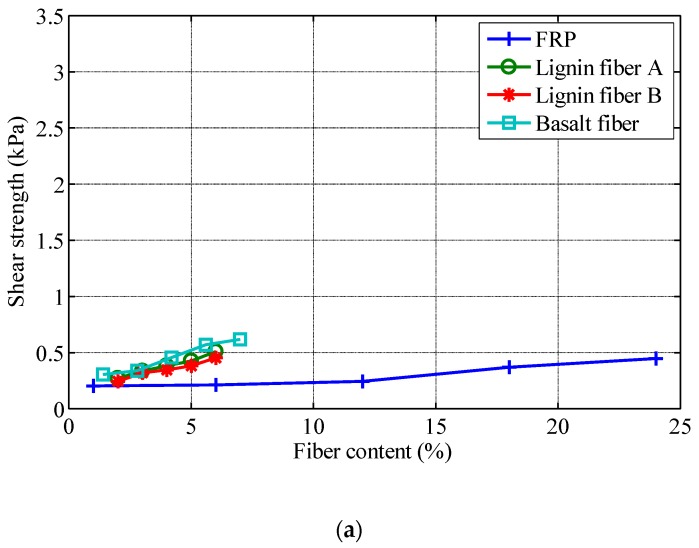

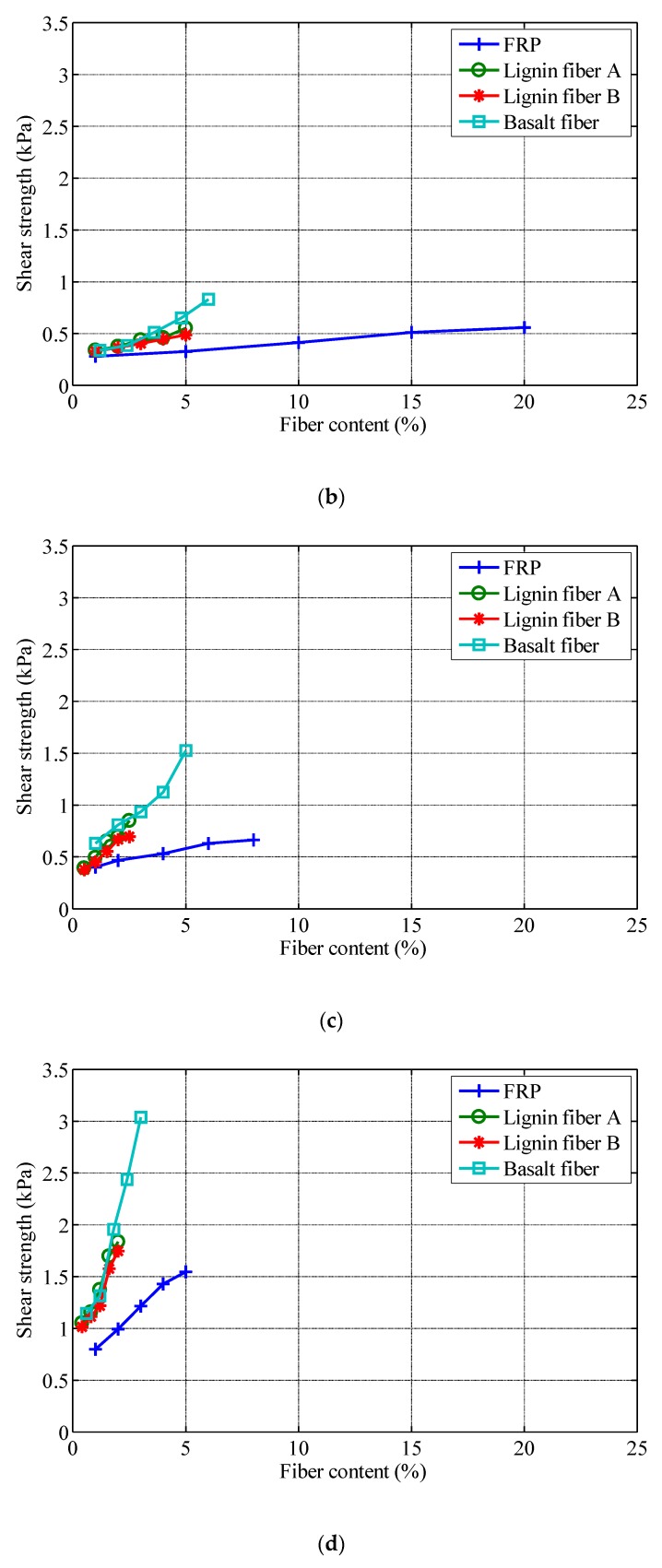
Shear strength of fiber-reinforced asphalt with various fiber contents: (**a**) asphalt No. 90; (**b**) asphalt No. 70; (**c**) styrene-butadiene-styrene (SBS) modified asphalt; (**d**) asphalt rubber.

In this study, the fall cone method is used to test the shear strength of asphalt and fiber-reinforced asphalt. The average values of three tests are used as test results. The shear strength of four kinds of asphalt is listed in the Table 7. For fiber-reinforced asphalt, the shear strength of fiber-reinforced asphalt with 2% content (fiber mass by asphalt mass) is tested first, and the results are also listed in Table 7. In order to investigate the effect of fiber content on the shear strength of fiber-reinforced asphalt, for various fiber-asphalt combinations, shear strength tests of fiber-reinforced asphalt with different fiber contents are carried out. Fiber content is gradually increased until agglomeration occurs. Figure 3 presents the typical normal fiber-reinforced asphalt surface and the agglomerated fiber-reinforced asphalt surface. Figure 4 depicts the shear strength of fiber-reinforced asphalt with various fiber contents (fiber mass by asphalt mass). It should be noted that the data point on the right side of each curve in Figure 4 corresponds to the agglomeration of fiber in asphalt.

It can be seen from Table 7 that asphalt rubber has the highest shear strength among the four asphalts, followed by SBS modified asphalt, asphalt No.70, and asphalt No.90. The shear strength of various asphalts increases with the addition of fiber. When the content of fiber is 2%, the shear strength of Basalt fiber-reinforced asphalt is the highest, followed by lignin fiber A, lignin fiber B, and FRP.

As can be seen in Figure 4, FRP can be added in the largest amount in the four test asphalts, followed by basalt fiber. The amounts of the two kinds of lignin fibers are significantly lower than that of the other two fibers. For the reinforcement effect, the shear strength of basalt fiber reinforced asphalt is higher than that of the other three fibers, and the reinforcement effect of two lignin fibers is similar to that of basalt fiber at the same content. However, the amount of basalt fiber that can be added is higher than that of two lignin fibers, so that basalt fiber can get a better reinforcement effect. The shear strength of FRP reinforced asphalt is significantly lower than that of the other three fibers, although the FRP content that can be added is significantly higher than that of the other three fibers. Even at high dosage, the FRP reinforced asphalt does not have better shear strength than the other three fibers and it has the worst strengthening effect among the four fibers.

By comparing the curve slope in Figure 4, it illustrates that the slope of shear strength curves for asphalt rubber is significantly higher than those for the other three asphalts with the same fiber. The results show that the fiber reinforcement effect is most significant in asphalt rubber, followed by SBS modified asphalt, and the fiber reinforcement effect is worse in asphalt No. 70 and asphalt No. 90.

## 4. Relationship between Interface Properties and Shear Strength

**Figure 5:**
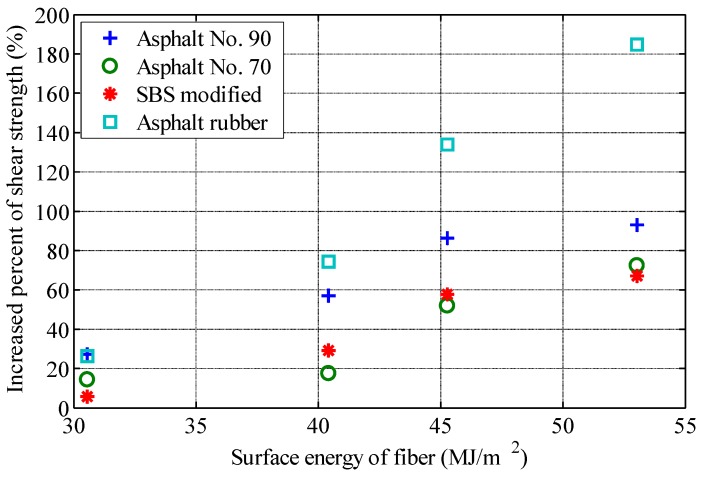
Relationship between surface energy of fiber and increase percent of shear strength.

**Figure 6:**
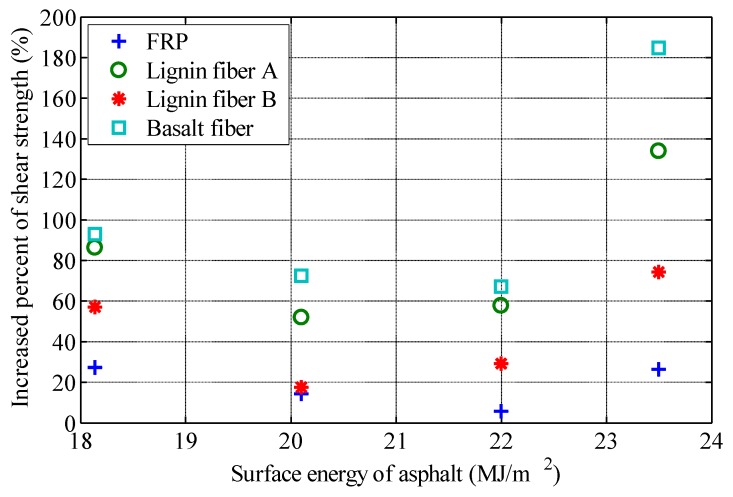
Relationship between surface energy of asphalt and increase percent of shear strength.

**Figure 7:**
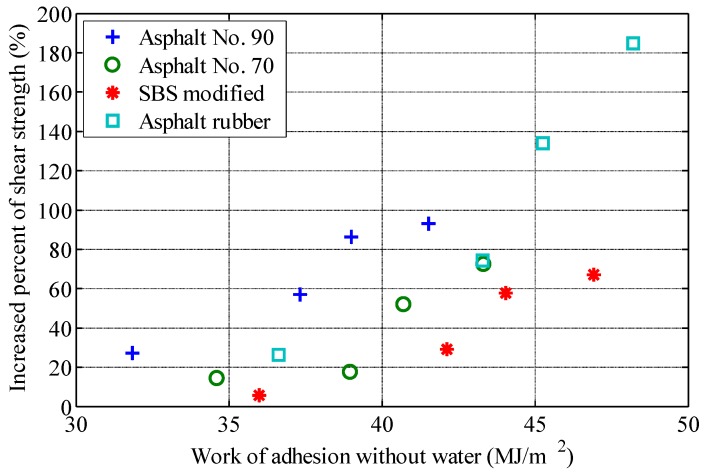
Relationship between work of adhesion and increase percent of shear strength.

In order to analyze the relationship between interface properties and fiber reinforcement properties, the increase percent of shear strength (IPSS) of fiber-reinforced asphalt with 2% content (fiber mass by asphalt mass) is calculated with reference to the shear strength of asphalt without fiber. The surface energy of fiber, the surface energy of asphalt, and the work of adhesions are plotted, respectively. The relationship between interface characteristic parameters and IPSS are shown in Figure 5, Figure 6 and Figure 7.

Figure 5 shows that IPSS increases with the increase of fiber surface energy for the same asphalt. Table 8 lists the Pearson correlation coefficients between IPSS and fiber surface energy of the four asphalts. All Pearson correlation coefficients are above 0.91, indicating that IPSS has a good correlation with fiber surface energy. However, Figure 5 also shows significant differences between different asphalts, indicating that the reinforcement effect of fiber changes significantly with different asphalts, and that the surface energy of fiber cannot be used solely to evaluate the reinforcement effect.

Figure 6 shows that for the same fiber, the relationship between IPSS and asphalt surface energy is approximately parabolic. When the asphalt surface energy is small, IPSS decreases with an increase of asphalt surface energy. As the asphalt surface energy continues to increase, IPSS begins to increase with an increase of asphalt surface energy. Table 9 lists the Pearson correlation coefficients between IPSS and asphalt surface energy corresponding to four kinds of fibers. All Pearson correlation coefficients are below 0.6, and the correlation between IPSS and asphalt surface energy is poor.

Figure 7 shows that IPSS increases with an increase of work of adhesion between asphalt and fiber for the same asphalt. Although the work of adhesion between asphalt and fiber reflects the interrelationship between asphalt and fiber, the relationship between work of adhesion and IPSS still shows a significant dependence on the types of asphalt, which indicates that the work of adhesion between asphalt and fiber cannot fully describe the difference of fiber reinforcement characteristics between different asphalts.

## 5. Discussion

It is a usual approach to reinforce asphalt using fibers for enhancing its performance. The interface properties of fibers and asphalts are investigated in this paper, which brings some new understanding of the mechanism of fiber-reinforced asphalt. Some researchers have evaluated the shear strength of fiber-reinforced asphalt using the fall cone method. Basalt fiber shows better reinforcing effect than lignin and other fibers for the same asphalt [28,29], which is similar to the findings in this paper. The higher surface energy of basalt fiber could be an explanation for its good reinforcing effect.

There are still some phenomena observed in this study that cannot be well explained. As shown in Figure 4, the FRP can be added in a large amount without agglomeration, which is several times more than that of the other 3 fibers. This phenomenon cannot be explained only in accordance with the interface properties of fibers and asphalts. Other properties, such as the specific surface area of the fibers, should also be evaluated for further investigation. For the same fiber, the reinforcing effect varies from asphalt to asphalt, which does not show a close relationship with the interface properties of asphalts. The influence of asphalt components should also be investigated in the future study.

## 6. Summary and Conclusion

In order to study the effect of interfacial properties on the performance of fiber-reinforced asphalt, four types of asphalt and four types of fibers are selected for laboratory tests. The surface energy of each kind of asphalt and fiber is measured, and the work of adhesion between asphalt and fiber is calculated. The shear strength of various asphalts and fiber-reinforced asphalts is measured by the fall cone method, and the interfacial properties are analyzed on the basis of the experimental measurements. The influences of the interfacial properties on the performance of fiber-reinforced asphalt are summarized as follows.

(1) The shear strength of asphalt binder can be increased significantly by the addition of fiber, which is closely related to the types of asphalt and fiber. For the same asphalt, basalt fiber has the best reinforcement effect among the four fibers, followed by the two lignin fibers, and the FRP. For the same fiber, asphalt rubber has been the most effectively reinforced, followed by the SBS modified asphalt, asphalt No.70 and asphalt No.90;
(2) For the same asphalt, there is a good positive correlation between the surface energy of fiber and the performance of fiber-reinforced asphalt. The higher the surface energy of fiber, the better the effect of fiber-reinforced asphalt. There is a good correlation between the work of adhesion between asphalt and fiber and the effect of fiber reinforcement, but there are still significant differences according to the type of asphalt;
(3) This study presented a basis for material selection in performance-oriented fiber-reinforced asphalt design. It can be concluded that fibers can be selected according to surface energy. High surface energy fibers have good reinforcement effect.

## Figures and Tables

**Table 1:** Parameters of the reference liquids for determining asphalt surface energy (MJ/m^2^).

Liquid	$\gamma_{L}$	$\gamma_{L}^{p}$	$\gamma_{L}^{d}$
Deionized water	72.8	51	21.8
Ethylene glycol	48	19	29
Glycerol	63.4	26.4	37
Formamide	58.2	18.7	39.5

**Table 2:** Parameters of the reference liquids for determining fiber surface energy.

Liquid	$\gamma_{L}$ (MJ/m^2^)	$\gamma_{L}^{LW}$ (MJ/m^2^)	$\gamma_{L}^{AB}$ (MJ/m^2^)	$\gamma_{L}^{+}$ (MJ/m^2^)	$\gamma_{L}^{-}$ (MJ/m^2^)	η (Pa·s)
Positive ethane	18.4	18.4	0	0	0	3
Diiodomethane	50.8	50.8	0	0	0	28
Toluene	28.3	28.3	0	0	2.7	6
Trichloromethane	27.3	27.3	0	3.8	0	5

**Table 3:** Contact angle between the asphalts and the reference liquids at 25 °C.

Asphalt Binder	Deionized Water	Ethylene Glycol	Glycerol	Formamide
Asphalt No. 90	107.45	84.94	93.54	88.48
Asphalt No. 70	101.60	81.82	90.99	85.63
SBS modified	102.15	77.55	87.39	81.92
Asphalt rubber	101.87	81.98	91.08	87.02

**Table 4:** Surface energy of the asphalt binders at 25 °C (MJ/m^2^).

Asphalt Binder	$\gamma_{A}$	$\gamma_{A}^{d}$	$\gamma_{A}^{p}$
Asphalt No. 90	18.138	17.435	0.704
Asphalt No. 70	20.099	19.379	0.720
SBS modified	21.999	20.932	1.068
Asphalt rubber	23.497	22.412	1.085

**Table 5:** Surface energy of the fibers (MJ/m^2^).

Fiber	$\gamma_{F}$	$\gamma_{F}^{LW}\;(\gamma_{F}^{d})$	$\gamma_{F}^{AB}\;(\gamma_{F}^{p})$
FRP	30.554	20.119	10.436
Lignin fiber A	45.278	32.908	12.370
Lignin fiber B	40.419	28.942	11.477
Basalt fiber	53.023	39.967	13.056

**Table 6:** Work of adhesion of asphalt-fiber interface without water at 25 °C (MJ/m^2^).

Fiber	Asphalt No. 90	Asphalt No. 70	SBS Modified	Asphalt Rubber
FRP	31.840	34.596	35.980	36.620
Lignin fiber A	38.996	40.702	44.041	45.253
Lignin fiber B	37.317	38.958	42.120	43.285
Basalt fiber	41.515	43.32	46.919	48.203

**Table 7:** Shear strength of asphalts without fiber and with 2% fiber (kPa).

Asphalt Binder	No Fiber	FRP	Lignin Fiber A	Lignin Fiber B	Basalt Fiber
Asphalt No. 90	0.17122	0.21782	0.31890	0.26896	0.33045
Asphalt No. 70	0.24628	0.28150	0.37403	0.28936	0.42458
SBS modified	0.43842	0.46390	0.69185	0.56665	0.73261
Asphalt rubber	0.78458	0.99179	1.83520	1.36836	2.23454

**Table 8:** Pearson correlation coefficient between surface energy of fiber and increase percent of shear strength (IPSS).

Asphalt	Asphalt No. 90	Asphalt No. 70	SBS Modified	Asphalt Rubber
Correlation coefficient	0.9672	0.9199	0.9722	0.9864

**Table 9:** Pearson correlation coefficient between surface energy of asphalt and increase percent of shear strength (IPSS).

Fiber	FRP	Lignin Fiber A	Lignin Fiber B	Basalt Fiber
Correlation coefficient	−0.1966	0.4616	0.2643	0.5885
